# Cost-effectiveness analysis of tislelizumab plus chemotherapy versus placebo plus chemotherapy as first-line treatment for extensive-stage small cell lung cancer in China

**DOI:** 10.3389/fpubh.2025.1652917

**Published:** 2025-10-14

**Authors:** Xiongxiong Fan, Zhengxiong Li, Dong Liu

**Affiliations:** ^1^Clinical Pharmacy Office, Baoji Central Hospital, Baoji, China; ^2^School of Medical Informatics and Engineering, Xuzhou Medical University, Xuzhou, China

**Keywords:** tislelizumab, extensive-stage small cell lung cancer, partitioned survival model, Markov, cost-effectiveness analysis

## Abstract

**Objective:**

The RATIONALE-312 trial demonstrated that the combination of tislelizumab with chemotherapy significantly improved the survival benefits for patients with extensive-stage small cell lung cancer (ES-SCLC). In this study, we used two models to evaluate the cost-effectiveness of tislelizumab in combination with chemotherapy as a first-line treatment for ES-SCLC patients from the perspective of China’s healthcare system.

**Methods:**

Based on the RATIONALE-312 trial data, a Markov model and a partitioned survival (PS) model were developed to assess the cost-effectiveness of tislelizumab in combination with chemotherapy as first-line treatment for ES-SCLC. The models set a 3-week cycle length and 10-year time horizon. Cost and utility values were obtained from the drug data service platform and published studies. Primary outcomes measured in the models included total costs, life-years (LYs), quality-adjusted life-years (QALYs) and incremental cost-effectiveness ratios (ICERs). Furthermore, one-way and probabilistic sensitivity analyses (PSA) were conducted to verify robustness of the models.

**Results:**

In the base-case analysis, the ICERs based on the Markov model and the PS models of tislelizumab group were CNY 216,041.10/QALY and CNY 206,915.66/QALY, respectively, compared with placebo group. One-way sensitivity analysis showed that the most influential parameters on the ICER were the utility of progression-free survival, and the cost of etoposide. The PSA indicated that at the current willingness-to-pay (WTP) threshold of CNY 268,074 per QALY, the probability of tislelizumab being cost-effective in two models was 93.60 and 86.70%, respectively.

**Conclusion:**

At the current WTP threshold in China, the combination of tislelizumab and chemotherapy may be cost-effective as a first-line treatment for patients with ES-SCLC.

## Introduction

Lung cancer is the malignant tumor with the highest morbidity and mortality in China (1), among which small cell lung cancer (SCLC) accounts for 15% of all lung cancer cases, and is characterized by strong invasiveness, rapid progression, easy early metastasis and poor prognosis (2, 3). 80 to 85% of patients are often in the extensive stage (ES-SCLC) when diagnosed, and the survival rate has decreased significantly, and the 2-year survival rate is even less than 15% (4, 5), which is a major clinical problem.

For 40 years, traditional platinum-based chemotherapy has been the standard first-line treatment for ES-SCLC. In recent years, the rise of immunotherapy has brought new hope for the treatment of ES-SCLC (6, 7). ASTRO-005 study met its primary endpoint and found an overall survival (OS) benefit (8), while the KEYNOTE-604 study failed to show a significant improvement in OS (9). Therefore, the potential survival benefits of anti-PD-1 therapy plus chemotherapy as first-line treatment for ES-SCLC remain uncertain.

Tislelizumab, as the most indication of immune checkpoint inhibitors (ICIs) included in medical insurance in China, has been widely used in clinical practice. In addition to the treatment of advanced NSCLC, tislelizumab also has significant curative effect on SCLC. RATIONALE-312 (10) study observed that the objective response rate (ORR) of first-line treatment with tislelizumab combined with chemotherapy for ES-SCLC was 68%, the median progression-free survival (PFS) was 4.7 months, and the median OS was 15.5 months.

Currently, there is a lack of domestic research reports investigating the potential survival benefits and cost-effectiveness of incorporating tislelizumab into the traditional platinum-based chemotherapy regimen for treating ES-SCLC. Therefore, from the perspective of Chinese healthcare system, this study will establish Markov model and partitioned survival (PS) model to study the economic performance of tislelizumab combined with chemotherapy in first-line treatment of ES-SCLC in China, in order to provide reference and basis for medical insurance access in China.

## Materials and methods

### Patients and intervention

A total of 457 patients were enrolled in the study. These patients were aged 18 years or older, had histologically or cytologically confirmed ES-SCLC, and had not previously received any treatment. Additionally, they were required to have an Eastern Cooperative Oncology Group Performance Status (ECOG PS) of 1 or less, a life expectancy of at least 12 weeks, and adequate organ function. Eligible patients with ES-SCLC were randomly assigned in a 1:1 ratio to receive either tislelizumab or placebo. The tislelizumab and placebo groups underwent four induction treatments over 21-day cycles, which included a 200 mg intravenous infusion of tislelizumab or placebo every 21 days, respectively. This was combined with epirubicin (100 mg/m^2^ intravenously on days 1–3 of each 21-day cycle) and platinum [either cisplatin (75 mg/m^2^) or carboplatin (AUC = 5), both administered intravenously on day 1 of each 21-day cycle]. From the fifth cycle onwards, 200 mg of tislelizumab or placebo was administered as maintenance therapy. After disease progression, based on data from the RATIONALE-312 study, it was assumed that 55% of patients in the tislelizumab group and 67% of patients in the placebo group would receive carboplatin or cisplatin plus etoposide as subsequent treatment. Furthermore, all patients in both groups were assumed to continue receiving best supportive care (BSC) regimens until death.

### Model overview

**Figure 1:**
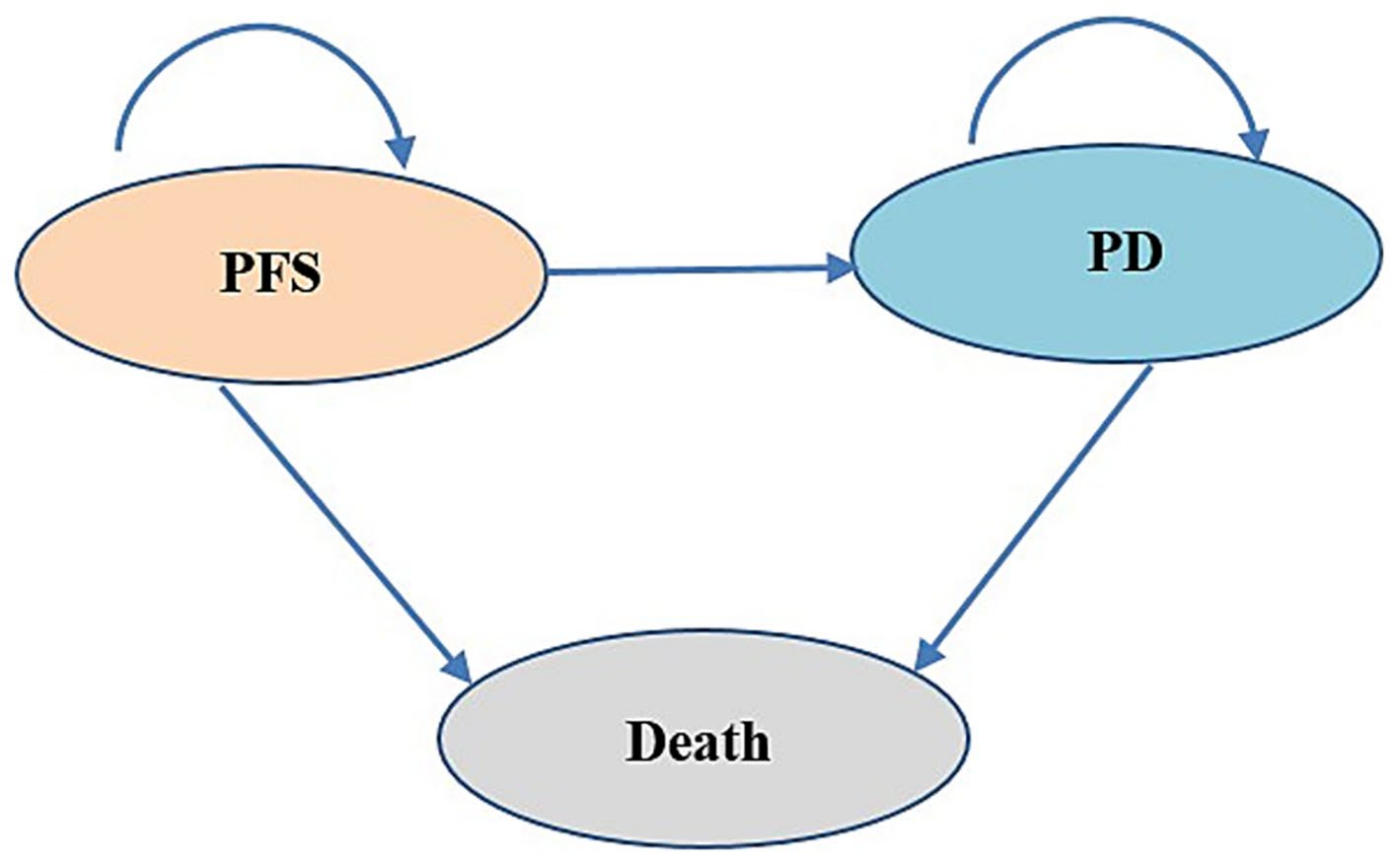
Model structure.

In this study, we utilized TreeAge Pro software to construct both the Markov model and the PS model to evaluate the cost-effectiveness of tislelizumab as a first-line treatment for patients with ES-SCLC. These two models were widely used in cost-effectiveness analysis. The model structure is shown in Figure 1. The models encompass three distinct, mutually exclusive health states: PFS, progressive disease (PD), and death. In the Markov model, transitions between health states are determined by transition probabilities. In the PS model, the proportion of PFS state patients was directly derived from PFS curve, while the proportion of patients in the death state was calculated as 1 minus OS curve. As for the PD state, its proportion was determined by the difference between the PFS and OS curves. One advantage of PS model was its ability to avoid estimating transition probability, making it easier to construct and calculate compared to Markov model. The duration for both models was set at 3 weeks according to patient dosing regimens, and the time horizon was 10 years. The total cost, life-years (LYs), quality-adjusted life-years (QALYs), and incremental cost-effectiveness ratio (ICER) were calculated. According to the China Guidelines for Pharmacoeconomic Evaluations (2020) (11), the willingness-to-pay (WTP) threshold was set at 3 times of China’s per capita GDP in 2023 (CNY 268,074/QALY). Both costs and health outcomes were discounted at an annual discount rate of 5%.

### Clinical data

We used WebPlotDigitizer (Version 4.6, https://automeris.io/WebPlotDigitizer/) from published RATIONALE-312 trail of PFS and OS extract data survival curves. The survHE package of R software (Version 4.2.2, https://www.r-project.org/) was used for data reconstruction. Exponential, Gamma, Generalized gamma, Gompertz, Weibull, Log-logistic and Log-lognormal distribution parameter models were utilized to fit and extrapolate long-term survival curves (Supplementary Figures S1, S2), respectively. The optimal fitting distribution was selected based on the values of Akaike Information Criterion (AIC), Bayesian Information Criterion (BIC) (Supplementary Table 1), and visual inspection. Finally, the OS and PFS curves of the tislelizumab group and the placebo group in this study were constructed using Log-logistic distribution, and the specific distribution parameters are shown in Table 1.

**Table 1:** Base-case key model inputs.

Parameter	Baseline value	Range	Distribution	Sources
Survival model of tislelizumab group				
Log-logistic model for OS	Scale (α) = 16.50756; Shape (*β*) = 1.70402		-	Model fitting
Log-logistic model for PFS	Scale (α) = 5.6371; Shape (β) = 1.81906		-	Model fitting
Survival model of placebo group				
Log-logistic model for OS	Scale (α) = 14.9796; Shape (β) = 1.9698		-	Model fitting
Log-logistic model for PFS	Scale (α) = 4.94181; Shape (β) = 2.51144		-	Model fitting
Cost per cycle (CNY)				
Tislelizumab	2,507.06	2,507.06 ~ 2,755.00	Gamma	www.yaozh.com
Cisplatin	102.81	82.22 ~ 641.13	Gamma	www.yaozh.com
Carboplatin	521.03	132.30 ~ 1,420.00	Gamma	www.yaozh.com
Etoposide	1,630.56	40.20 ~ 3,250.16	Gamma	www.yaozh.com
Best supportive care	2,467.00	1,973.65 ~ 2,960.47	Gamma	(25)
Routine follow-up	600.58	480.46 ~ 720.70	Gamma	(25)
Cost of AEs per unit				
Neutropenia	607.45	485.92 ~ 728.91	Gamma	(25)
White blood cell count decreased	3,361.50	2,689.19 ~ 4,033.79	Gamma	(25)
Thrombocytopenia	7,603.00	6,082.42 ~ 9,123.64	Gamma	(25)
Anemia	3,665.90	2,932.73 ~ 4,399.07	Gamma	(25)
Risk of AEs in tislelizumab group				
Neutropenia	0.5595	0.504 ~ 0.615	Beta	(10)
White blood cell count decreased	0.2379	0.214 ~ 0.262	Beta	(10)
Thrombocytopenia	0.1938	0.174 ~ 0.213	Beta	(10)
Anemia	0.1630	0.147 ~ 0.179	Beta	(10)
Risk of AEs in placebo group				
Neutropenia	0.5459	0.491 ~ 0.600	Beta	(10)
White blood cell count decreased	0.2751	0.248 ~ 0.303	Beta	(10)
Thrombocytopenia	0.2533	0.228 ~ 0.279	Beta	(10)
Anemia	0.1659	0.149 ~ 0.182	Beta	(10)
Proportion of subsequent chemotherapy				
Tislelizumab group	0.55	0.495 ~ 0.605	Beta	(10)
Placebo group	0.67	0.603 ~ 0.737	Beta	(10)
Utility				
PFS	0.804	0.724 ~ 0.884	Beta	(12)
PD	0.321	0.289 ~ 0.353	Beta	(12)
Neutropenia	0.200	0.180 ~ 0.220	Beta	(12)
White blood cell count decreased	0.200	0.180 ~ 0.220	Beta	(12)
Thrombocytopenia	0.190	0.171 ~ 0.209	Beta	(12)
Anemia	0.078	0.070 ~ 0.086	Beta	(12)
Others				
Discount rate (%)	5	0 ~ 8	Fixed in PSA	(11)

### Cost and utility parameters

In this study, the Chinese healthcare system was selected as the research perspective, so only direct medical costs were considered, including the costs of different treatment options, the costs after disease progression, the costs of BSC, the costs of adverse events (AEs) and follow-up costs. The drug cost data we used were obtained from a public database (https://www.yaozh.com/) and were the median bid prices for drug procurement by Chinese provinces in 2023. Assume that the average body weight of the patient is 65 kg and the body surface area was 1.72m^2^ (17). In this study, the cost of grade 3 ~ 4 AEs (≥5%) of RATIONALE-312 were taken into account and calculated at in initial cycle of the models. Health utility value was not reported in the RATIONALE-312 study, so utility parameters in this study are mainly reported from published literatures (12), indicating that the utility of PFS is 0.804 and the state utility of PD is 0.321. In addition, we also considered the disutility caused by AEs of grade 3 or above. Key inputs of all costs and utilities are shown in Table 1.

### Sensitivity analyses

In this study, one-way sensitivity analysis (OWSA) and probabilistic sensitivity analysis (PSA) were used to evaluate the uncertainty of the model. OWSA calculates ICER with the maximum and minimum values of the parameters or 95% confidence interval (CI) as the upper and lower limits to determine the extent of the impact of different parameters on the ICER when changing within a certain range. We selected the highest and lowest bid prices in various regions of the country, and the remaining costs were taken as the upper and lower limits with ±20% of the base-case value, and the utility value and the probability of AEs were changed with ±10% of the baseline value. The OWSA results were presented by the tornado diagram. The distribution of each parameter was sampled by PSA through 1,000 second-order Monte Carlo simulations, in which the cost parameter was gamma distribution, the utility value and the probability parameters were beta distribution, and the results were presented as an incremental cost-effectiveness scatter plot and cost-effectiveness acceptability curves (CEACs).

## Results

### Base-case analysis

The base-case analysis results are shown in Table 2. In the PS model, the total cost for the tislelizumab group was CNY 166,499.82, achieving 1.92 LYs and 0.76 QALYs. In comparison, the placebo group incurred a total cost of CNY 124,844.87 and gained 1.65 LYs and 0.56 QALYs. Compared to the placebo group, the tislelizumab group provided an additional 0.20 QALYs at an incremental cost of CNY 41,654.95, resulting in an ICER of CNY 206,915.66 per QALY gained. In the Markov model, the tislelizumab group had a total cost of CNY 166,244.56 with a health benefit of 1.91 LYs and 0.76 QALYs, while the placebo group had a total cost of CNY 125,867.48 with a gain of 1.67 LYs and 0.57 QALYs. The incremental cost for tislelizumab versus placebo was CNY 40,377.08, and the incremental QALYs were 0.19, resulting in an ICER of CNY 216,041.10 per QALY gained. The ICERs calculated in two models are lower than a WTP threshold of CNY 268,074/QALY. Therefore, tislelizumab can be considered cost-effective at the WTP threshold.

**Table 2:** Results of cost-effectiveness analysis.

Model	Group	Total cost (CNY)	LYs	QALYs	ICER (CNY/QALY)
PS	Tislelizumab	166,499.82	1.92	0.76	206,915.66
	Placebo	124,844.87	1.65	0.56	-
Markov	Tislelizumab	166,244.56	1.91	0.76	216,041.10
	Placebo	125,867.48	1.67	0.57	-

### Sensitivity analyses

**Figure 2:**
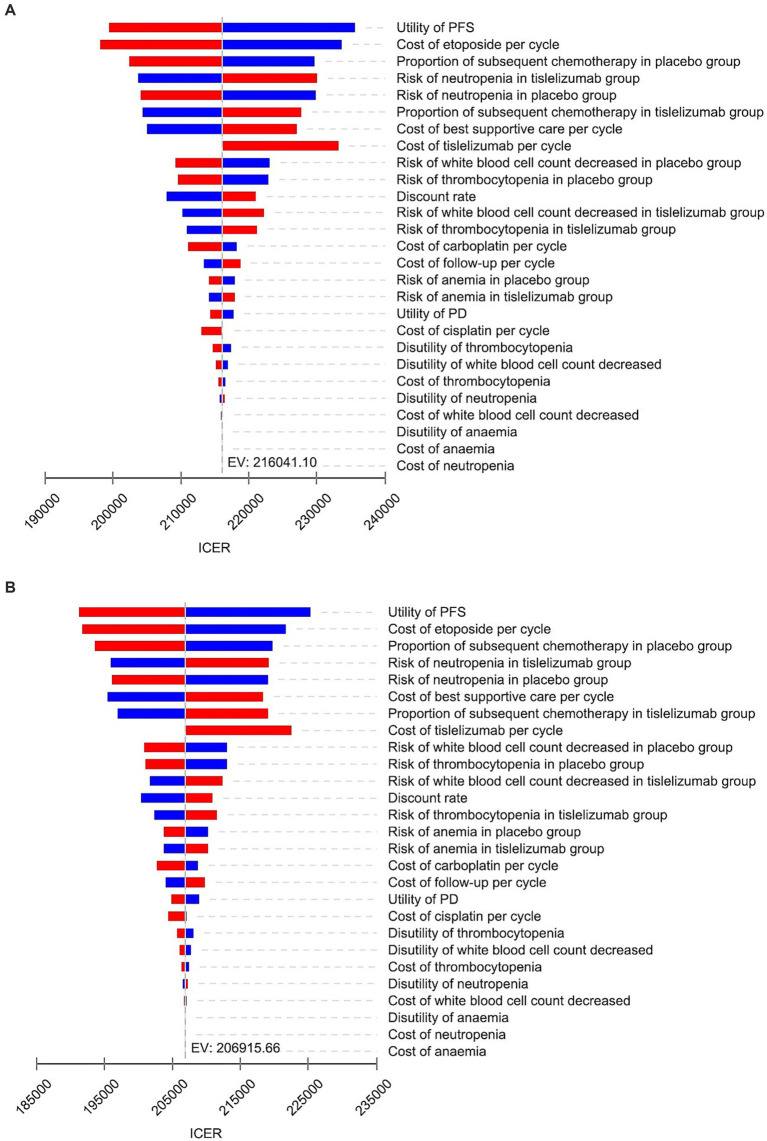
Tornado diagram of one-way sensitivity analysis. **(A)** Markov model. **(B)** PS model.

The results of OWSA are presented in Figure 2. Based on the two models, key parameters that significantly influenced the ICERs include the utility value of PFS, the cost of etoposide, proportion of subsequent chemotherapy in placebo group, the incidence of neutropenia in tislelizumab group, and cost of BSC. However, irrespective of individual parameter variations within the specified range in both models, the ICER value remained below three times China’s per capita GDP threshold, indicating consistent cost-effectiveness between tislelizumab and placebo group. These findings demonstrated robustness in our underlying analysis.

**Figure 3:**
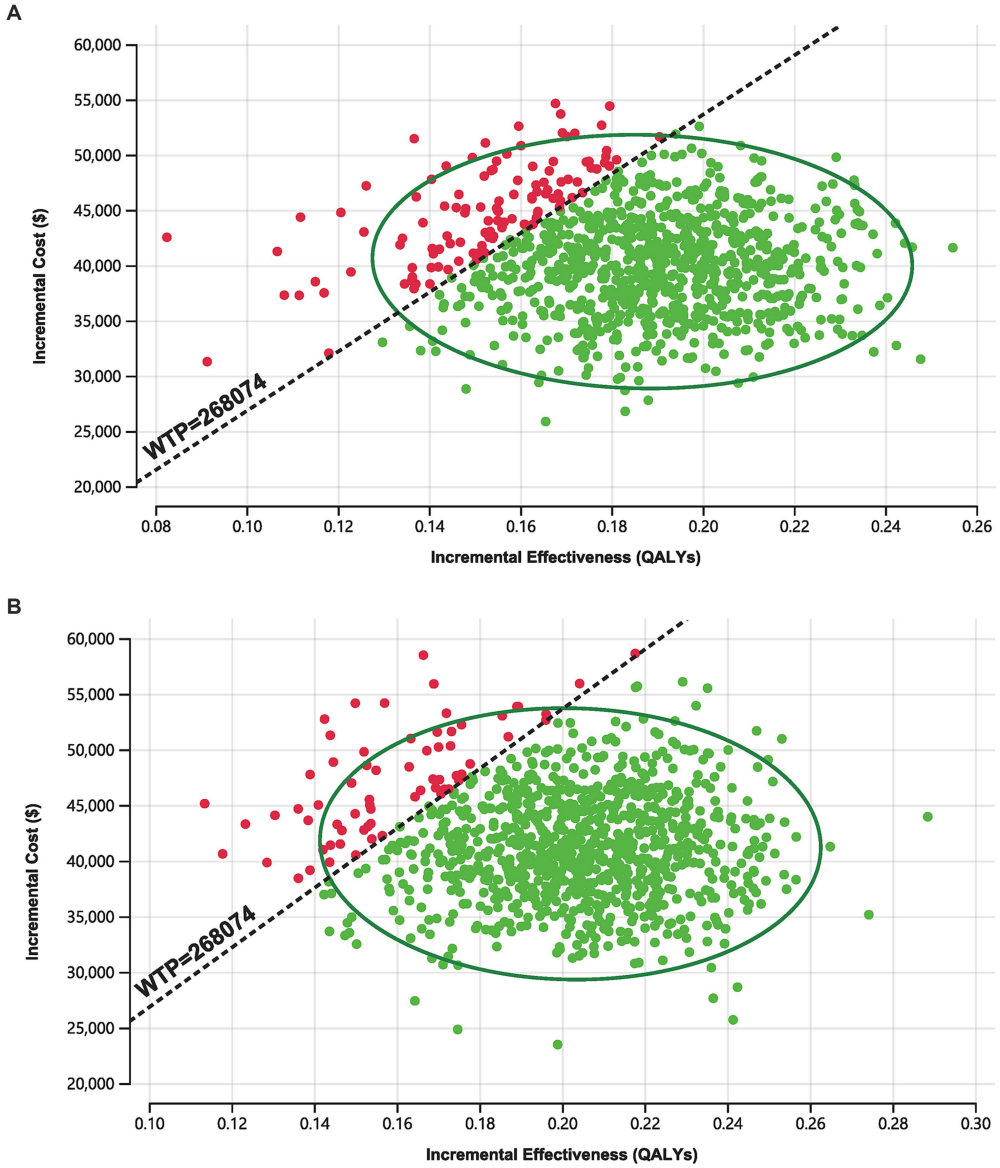
Incremental cost-effectiveness scatter plot. **(A)** Markov model. **(B)** PS model.

As shown in Figure 3, in the 1,000 Monte Carlo simulations, the majority of scatter points were concentrated within the 95% CI (represented by the ellipse). In the Markov model, approximately 86.7% of the scatter points lay below the WTP threshold line, while in the PS model approach, 93.60% of the scatter points were located below the WTP threshold line. In other words, the cost-effectiveness acceptability probabilities for tislelizumab in these two models were 86.70 and 93.60%, respectively.

**Figure 4:**
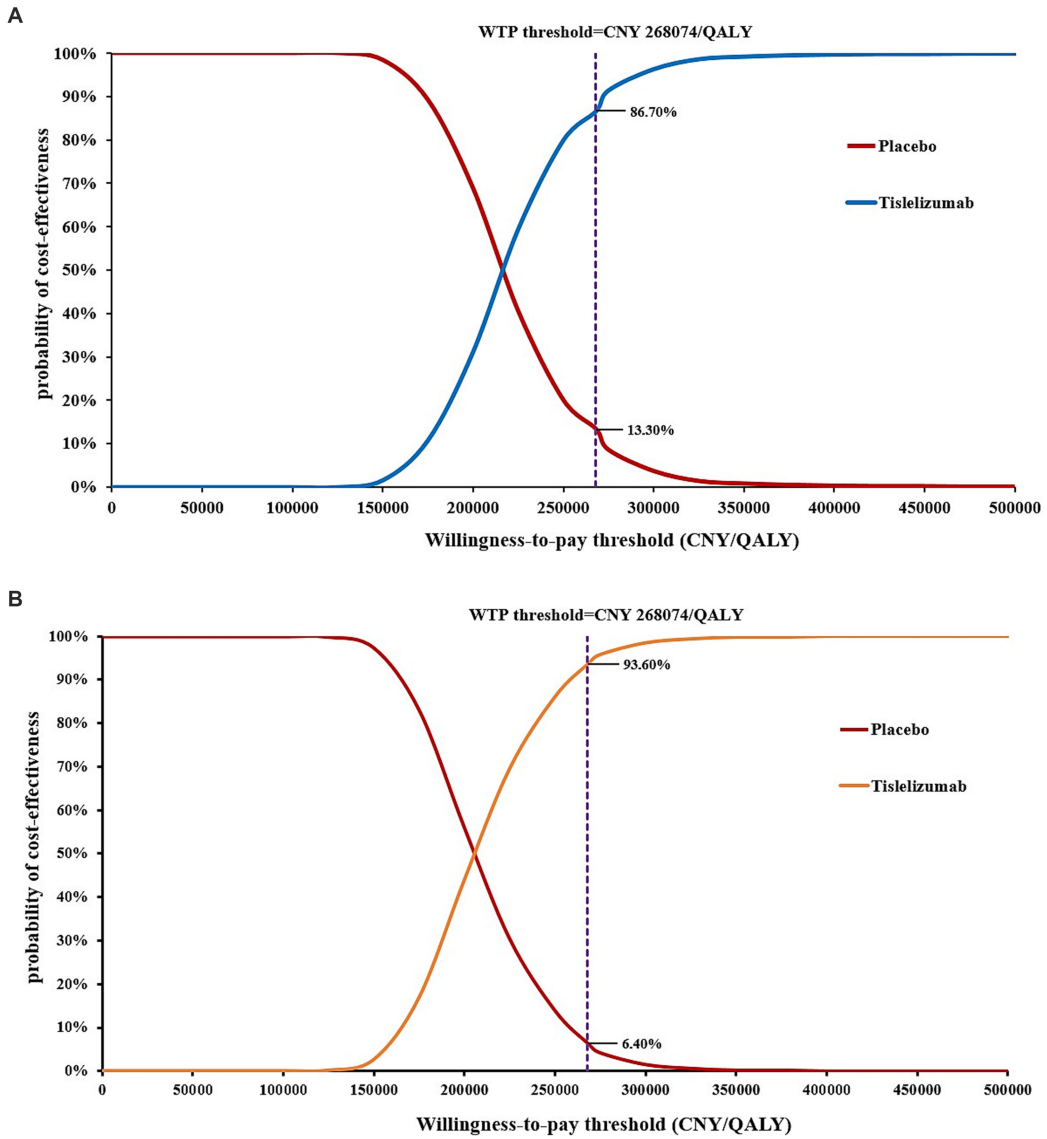
Cost-effectiveness acceptability curve. **(A)** Markov model. **(B)** PS model.

The CEACs are presented in Figure 4. According to the Markov model, when the WTP threshold was below CNY 124,922/QALY, the probability of tislelizumab group demonstrating cost-effectiveness was only 0.1%. However, at a WTP threshold of CNY 215,263/QALY, tislelizumab exhibited a substantial advantage with a probability of cost-effectiveness reaching 50% compared to placebo. Moreover, for WTP thresholds exceeding CNY 346,084/QALY, tislelizumab consistently demonstrated a full probability (100%) of cost-effectiveness. In the context of the PS model, when the WTP threshold was below CNY 117,684/QALY, the tislelizumab group exhibited a marginal probability of cost-effectiveness advantage compared to placebo group. Particularly, the likelihood of a cost-effectiveness advantage in the tislelizumab group at a WTP threshold of CNY 207,221/QALY was 50% and at WTP threshold exceeding CNY 352,517/QALY, tislelizumab consistently achieved a certain and significant probability (100%) of being cost-effective.

To summarize our findings collectively from various analytical perspectives employed throughout this study, it can be concluded that under identical WTP thresholds, the use of tislelizumab in patients yields significant economic benefits particularly within PS model analysis.

## Discussion

In this study, based on the RATIONALE-312 study, Markov and PS models were developed to compare the economic performance of both programs in patients with ES-SCLC, incorporating relevant price data from public database in China, aiming to investigate the cost-effectiveness of tislelizumab combined with chemotherapy as a first-line treatment for ES-SCLC. The primary findings of this study revealed that, in comparison to conventional chemotherapy, the ICERs for tislelizumab combined with chemotherapy were CNY 216,041.10/QALY and CNY 206,915.66/QALY in the two models respectively, both falling below WTP threshold. Sensitivity analyses confirmed the robustness of the model results, with both models yielding comparable outcomes. Although the outputs from the two models were not entirely identical, the conclusions drawn were consistently aligned. The above findings suggest that the combination of tislelizumab and chemotherapy demonstrates a substantial economic value in the first-line treatment of ES-SCLC within the framework of China’s healthcare system, prevailing drug prices, and study duration.

The application of ICIs marks a revolutionary change in the field of cancer treatment, opening a new path for multiple cancer therapies (13, 14). However, the exorbitant cost associated with immune checkpoint inhibitors poses a significant challenge to healthcare systems. For immunotherapy in ES-SCLC, the Chinese Society of Clinical Oncology Guidelines recommend combining chemotherapy with immunotherapy as the preferred treatment strategy. Notably, prominent ICIs include serpluliumab, atezolizumab, adebrelimab, and durvalumab. Nevertheless, pharmacoeconomic studies have demonstrated that utilizing these drugs in combination with chemotherapy as first-line therapy for ES-SCLC is not cost-effective when compared to chemotherapy alone in China (15–18). In 2024, toripalimab and benmelstobart were also granted approval for the treatment of ES-SCLC.

On June 28, 2024, the National Medical Products Administration (NMPA) of China granted approval for tislelizumab, a PD-1 inhibitor, in combination with etoposide and platinum-based chemotherapy as a first-line treatment option for patients diagnosed with ES-SCLC, based on clinical trial data from RATIONALE 312. Tislelizumab, an independently developed PD-1 inhibitor by Chinese pharmaceutical companies, offers significant advantages over imported immunotherapy agents in terms of reduced transportation costs and a greater price reduction compared to similar inhibitors. Consequently, tislelizumab presents a more accessible and widely applicable treatment option for Chinese patients.

As of October 2024, the PD-1/PD-L1 immunotherapy drugs mentioned above have not been included in China’s NMPA medical insurance reimbursement list for ES-SCLC. Consequently, patients are responsible for covering the expenses of these drugs as they are not eligible for reimbursement through the healthcare insurance system. Therefore, the localization of immunotherapy drugs has significantly mitigated the treatment expenses (19, 20). It is anticipated that in the foreseeable future, with the backing of diverse measures such as national medical insurance negotiations, there will be a substantial reduction in the therapeutic costs associated with ES-SCLC (21).

Both PS and Markov models entail inherent methodological limitations in pharmacoeconomic evaluations (22). The PS models approach relies heavily on long-term extrapolation of progression-free survival and overall survival curves, rendering results highly sensitive to the choice of extrapolation function. It also assumes independence between disease progression and death—a simplification that may not reflect clinical reality (23). In contrast, standard Markov models are bound by the “memoryless” property (24), meaning future transitions depend only on the current health state and not on the patient’s history, thereby limiting their ability to account for time-varying risks. Moreover, each model faces distinct structural challenges: PS models lack flexibility in representing complex disease trajectories and integrating time-dependent covariates, whereas Markov models are prone to “state explosion” as model complexity increases, and their accuracy hinges on transition probabilities that are often difficult to estimate and validate robustly.

The strengths of this study are as follows: Firstly, this economic evaluation is based on the final research data from RATIONALE-312, thereby reducing uncertainties associated with long-term effects and economic estimates observed in previous evaluations. Secondly, by integrating the analysis results of both Markov and PS models, this study enhances the reliability of cost-effectiveness findings.

This study also has some limitations. First, it should be noted that the perspective adopted in this research is specific to China’s healthcare system. Consequently, due to variations in costs, WTP, and discount rates across different countries, the findings of this study may not be directly applicable to other nations or alternative research perspectives. Second, we did not conduct subgroup analyses or further stratified evaluations, which could have provided more clinically nuanced insights. Third, it is important to acknowledge that the utility values employed in this investigation are derived from foreign studies as there is currently no available research on domestic ES-SCLC utility values. This may result in some deviations between the simulation results and the actual health outcomes. Fourth, in our model, we only considered the costs and disutility associated with grade 3–4 AEs, which may lead to some bias in the model outputs to a certain extent. Fifth, we assumed that BSC would be provided after disease progression, which may differ from the actual clinical treatment choices.

## Conclusion

In summary, from the perspective of China’s healthcare system and using three times China’s per capita GDP in 2023 as the WTP threshold, the combination of tislelizumab and chemotherapy may be a cost-effective treatment option in the first-line treatment of patients with ES-SCLC. This finding holds significant implications for both China’s healthcare system and clinical practice.

## Data Availability

The original contributions presented in the study are included in the article/Supplementary material, further inquiries can be directed to the corresponding author/s.
