# Supplementary material for: Cost-effectiveness analysis of tislelizumab plus chemotherapy versus placebo plus chemotherapy as first-line treatment for extensive-stage small cell lung cancer in China
Source: Front Public Health. 2025 Oct 14;13:1652917. doi: 10.3389/fpubh.2025.1652917 (PMC12558940; doi:10.3389/fpubh.2025.1652917)
Supplement: Supplementary file 1 [file Data_Sheet_1.pdf]

Cost-effectiveness analysis of tislelizumab plus chemotherapy versus placebo plus chemotherapy as first-line treatment for extensive-stage small cell lung cancer in China

Xiongxiong Fan[1]†, Zhengxiong Li[2]†, Dong Liu[1]

[1]From Clinical Pharmacy Office, Baoji Central Hospital, Baoji, Shaanxi, China

[2]School of Medical Informatics and Engineering, Xuzhou Medical University, Xuzhou, China

Correspondence to: Dong Liu, Clinical Pharmacy Office, Baoji Central Hospital, Baoji, Shaanxi, China, 8 Jiangtan Road, Weibin District, Baoji City, Shaanxi Province. E-mail: **liudong691122@126.com** (D. L.)

†These authors have contributed equally to this work.

Table S1. AIC and BIC statistics for alternate parametric distributions.

Parameter	AIC		BIC	
	Tislelizumab group	Placebo group	Tislelizumab group	Placebo group
OS curves				
Weibull (AFT)	1368.49	2827.53	1375.34	2835.78
Log-normal	1366.38	2815.25	1373.23	2823.50
Log-logistic	1356.76	2796.82	1363.61	2805.07
Gompertz	1377.39	2861.63	1384.24	2869.88
Generalized gamma	1364.21	2810.36	1374.48	2822.73
Gamma	1365.49	2816.35	1372.33	2824.60
Exponential	1376.57	2873.43	1380.00	2877.55
PFS curves				
Weibull (AFT)	1189.85	2358.61	1196.70	2366.86
Log-normal	1132.21	2200.01	1139.07	2208.26
Log-logistic	1115.20	2130.91	1122.05	2139.16
Gompertz	1160.35	2347.21	1167.20	2355.46
Generalized gamma	1129.55	2192.24	1139.83	2204.61
Gamma	1188.12	2324.08	1194.97	2332.33
Exponential	1188.04	2368.51	1191.47	2372.64

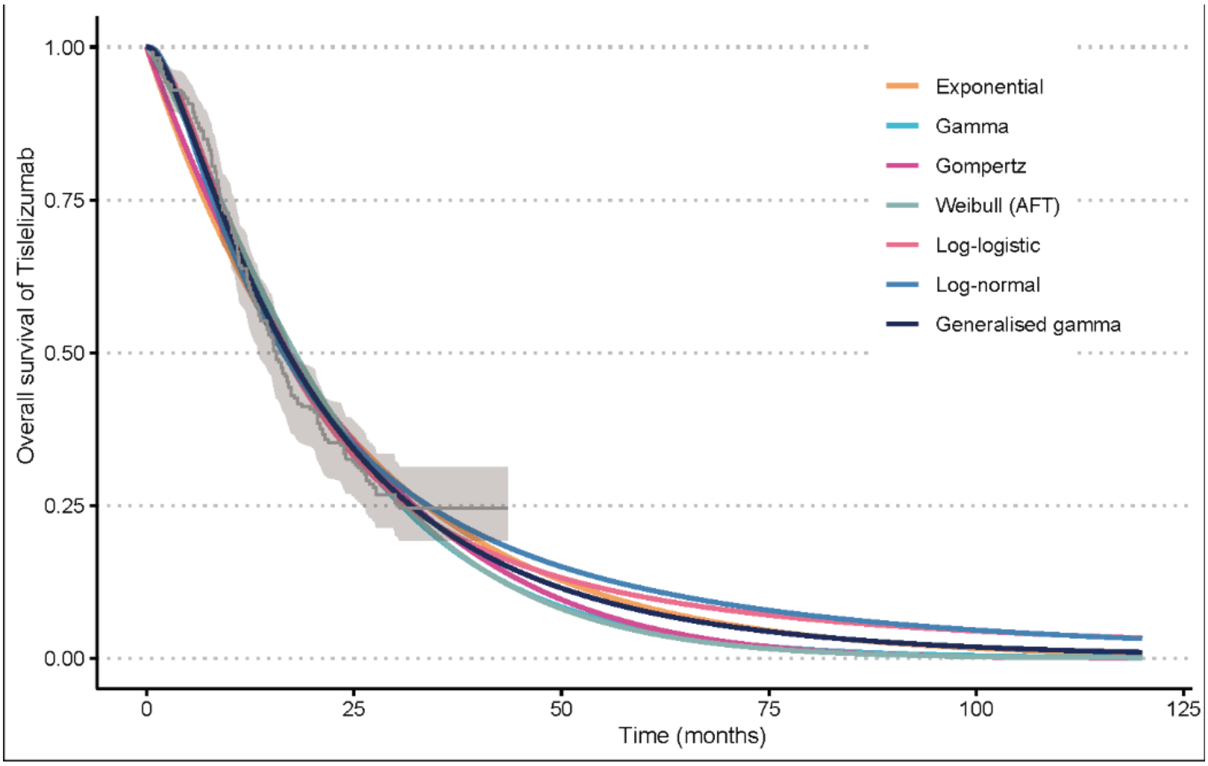

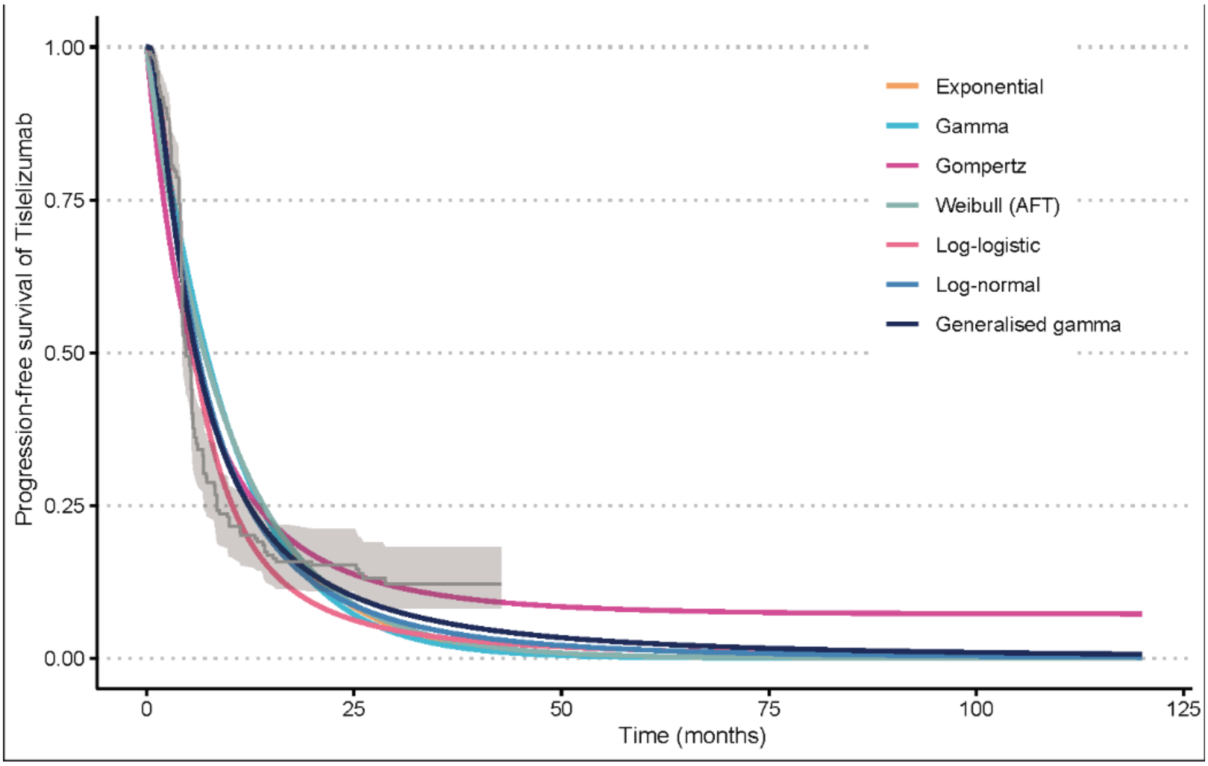

Figure S1. Kaplan-Meier curves fitting and extrapolation of tislelizumab plus chemotherapy group.

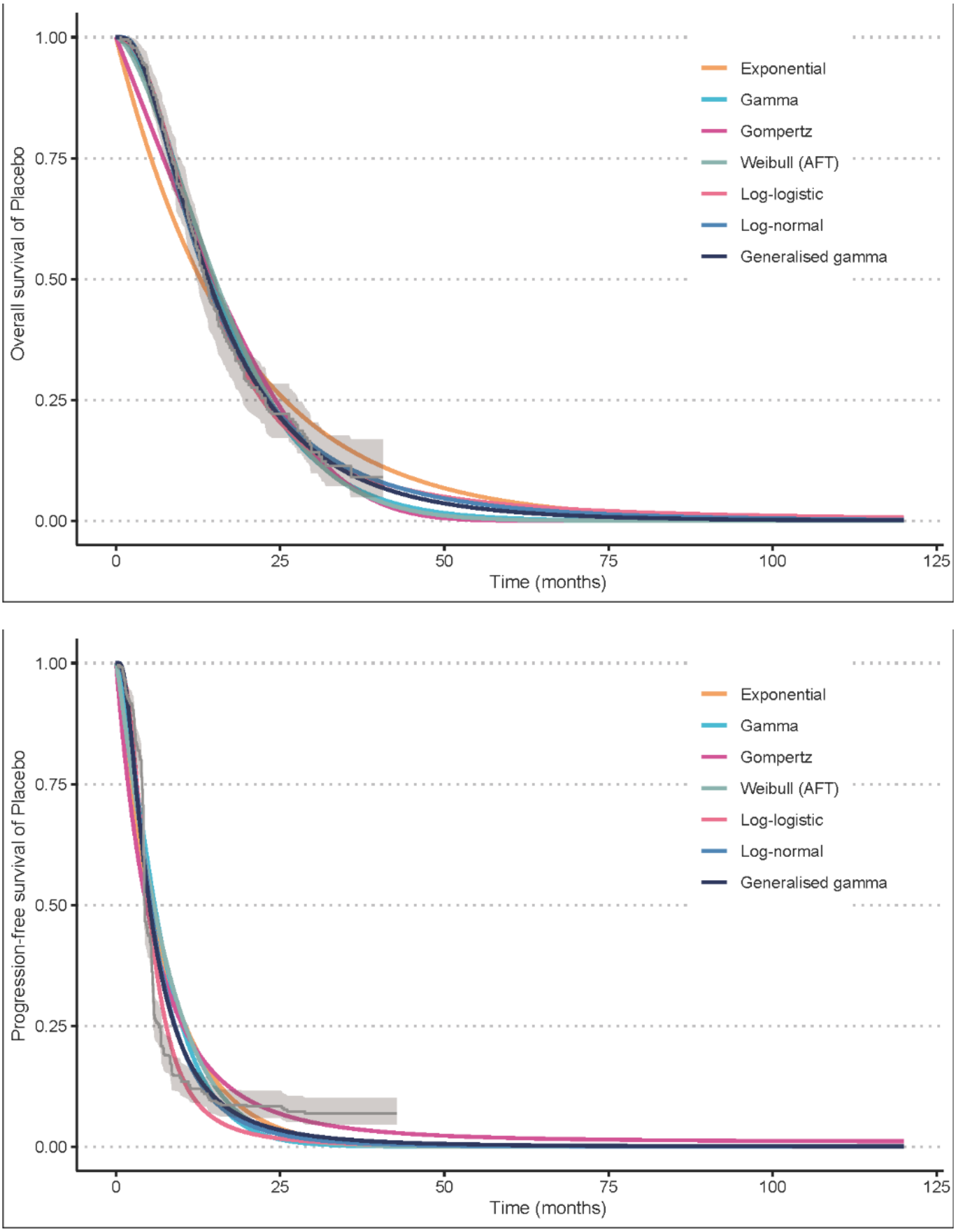

Figure S2. Kaplan-Meier curves fitting and extrapolation of placebo plus chemotherapy group.
